# Wildlife-livestock interactions and risk areas for cross-species spread of bovine tuberculosis

**DOI:** 10.4102/ojvr.v84i1.1221

**Published:** 2017-01-23

**Authors:** Natascha V. Meunier, Peregrine Sebulime, Richard G. White, Richard Kock

**Affiliations:** 1 Department of Pathology and Pathogen Biology, Royal Veterinary College, United Kingdom; 2 Faculty of Epidemiology and Population Health, London School of Hygiene and Tropical Medicine, United Kingdom; 3 Department of Wildlife and Aquatic Animal Resources, Makerere University, Uganda

## Abstract

The transmission of diseases between livestock and wildlife can be a hindrance to effective disease control. Maintenance hosts and contact rates should be explored to further understand the transmission dynamics at the wildlife-livestock interface. Bovine tuberculosis (BTB) has been shown to have wildlife maintenance hosts and has been confirmed as present in the African buffalo (*Syncerus caffer*) in the Queen Elizabeth National Park (QENP) in Uganda since the 1960s. The first aim of this study was to explore the spatio-temporal spread of cattle illegally grazing within the QENP recorded by the Uganda Wildlife Authority (UWA) rangers in a wildlife crime database. Secondly, we aimed to quantify wildlife-livestock interactions and cattle movements, on the border of QENP, using a longitudinal questionnaire completed by 30 livestock owners. From this database, 426 cattle sightings were recorded within QENP in 8 years. Thirteen (3.1%) of these came within a 300 m–4 week space-time window of a buffalo herd, using the recorded GPS data. Livestock owners reported an average of 1.04 (95% CI 0.97–1.11) sightings of Uganda kob, waterbuck, buffalo or warthog per day over a 3-month period, with a rate of 0.22 (95% CI 0.20–0.25) sightings of buffalo per farmer per day. Reports placed 85.3% of the ungulate sightings and 88.0% of the buffalo sightings as further than 50 m away. Ungulate sightings were more likely to be closer to cattle at the homestead (OR 2.0, 95% CI 1.1–3.6) compared with the grazing area. Each cattle herd mixed with an average of five other cattle herds at both the communal grazing and watering points on a daily basis. Although wildlife and cattle regularly shared grazing and watering areas, they seldom came into contact close enough for aerosol transmission. Between species infection transmission is therefore likely to be by indirect or non-respiratory routes, which is suspected to be an infrequent mechanism of transmission of BTB. Occasional cross-species spillover of infection is possible, and the interaction of multiple wildlife species needs further investigation. Controlling the interface between wildlife and cattle in a situation where eradication is not being considered may have little impact on BTB disease control in cattle.

## Introduction

Circumstantial evidence suggests that transmission of a range of diseases between wildlife and livestock occasionally occurs either directly or through a vector (Bengis, Kock & Fischer 2002; Miller, Farnsworth & Malmberg 2013; Siembieda et al. 2011). Transmission between species is dependent on the population distribution and timing of contacts (Renwick, White & Bengis 2007), and where weak inter-species transmission occurs, spread within species needs to be high for the disease to persist in the ecosystem. The wildlife-livestock interface has been well studied for bovine tuberculosis (BTB) in countries with intensive control programmes, and wildlife have been implicated as a reservoir of infection to cattle (Palmer 2013). Molecular studies indicate that isolates of BTB have been found in both domestic and wild animals supporting cross-species transmission, although the direction of transmission is not proven (De Garine-Wichatitsky et al. 2013; Musoke et al. 2015). Mathematical models are useful to estimate the contact rates and effective transmission between species, which can assist with the management of intervention strategies (Barron, Nugent & Cross 2013; Brooks-Pollock & Wood 2015; Hardstaff et al. 2013; Zanella et al. 2012). GPS collaring studies have also assisted with estimating these contact rates and probabilities of wildlife-livestock interactions over landscapes and time (Brook et al. 2013; Caron et al. 2016; Miguel et al. 2013).

In Uganda, African buffalo (*Syncerus caffer*) are maintenance hosts for BTB in the Queen Elizabeth National Park (QENP), with reports of the disease dating back to the 1960s (Woodford 1982a). Infection levels have not notably increased in buffalo since this time, with Kalema-Zikusoka et al. (2005) proposing that the drastic population decreases because of poaching in the 1970s and 1980s limited the spread of the infection. Alternately, the lack of fencing may allow buffalo to expand to other reserves according to natural resource fluctuations, maintaining lower density populations and limiting disease spread. Both cattle and buffalo numbers are increasing which may bring animals into closer contact because of grazing pressures and impact on potential cross-species interactions (Plumptre et al. 2010). Communities that keep livestock, within QENP and in neighbouring villages, are not fenced out of the park, and there is much anecdotal evidence of wildlife coming into contact with livestock. Because of limited grazing land, some farmers are known to drive their livestock illegally into QENP for extra forage, potentially coming into closer contact with wildlife (G. Kalule, pers. comm., November 2014).

The first aim of this study was to explore the spatio-temporal spread of cattle illegally grazing within the QENP, Uganda, recorded by the Uganda Wildlife Authority (UWA) rangers in a wildlife crime database. Secondly, we aimed to quantify wildlife-livestock interactions at the northern border of the QENP demarcated by the Nyamugasani River, using a longitudinal questionnaire.

## Materials and methods

### Ethics statement

All research activities in Uganda were reviewed and approved by UWA, the Uganda National Council for Science and Technology (UNCST) and the Royal Veterinary College Research Ethics Committee (URN201041290/ PPB01253).

### Study area

Our study site was QENP, a 1978 km^2^ wildlife protected area situated in western Uganda (00°12’S, 30°00’E) adjacent to the Democratic Republic of Congo and forms part of the Greater Virunga landscape, which is comprised of multiple wildlife reserves (Uganda Wildlife Authority 2012). Large herbivores present in QENP include African buffalo, Uganda kob (*Kobus kob thomasi*), defassa waterbuck (*Kobus ellipsiprymnus*), warthog (*Phacochoerus africanus*) and African elephant (*Loxodonta africana*). Predators include lion (*Panthera leo*), spotted hyena (*Crocuta crocuta*) and leopard (*Panthera pardus*). Livestock owners were from the region on the north-western border of the park and were predominantly Basongora pastoralists with a majority of Ankole cattle, an indigenous breed. There are no physical barriers preventing animal movement across the QENP borders except in the far south of the park.

### Wildlife crime database

The wildlife crime database was part of a UWA project that was undertaken in partnership with the Wildlife Conservation Society of New York. Rangers on daily patrols recorded illegal activities and animal sightings within the QENP, including grazing of domestic animals on park land. A data set was obtained in March 2015 from the UWA Department of Research and Monitoring containing GPS coordinates for buffalo and cattle sightings within the protected area. The sightings were recorded from January 2006 until the end of March 2014 with variable quality of reporting. Uganda kob were not recorded in this database as they were numerous and the task became burdensome to the field staff recording the data.

Spatio-temporal analysis was conducted to identify overlapping areas of buffalo and cattle sightings. Each sighting was representative of one herd on a specific day, and sightings were considered repeats if recorded within immediate proximity and time. Temporal limits were 2, 4 and 6 weeks based on the survival time of mycobacteria in the environment (Tanner & Michel 1999). Spatial limits were 150 m, 300 m and 500 m to take into account the variability of the GPS equipment and recording methods, and movement of animals around the recorded site. Any buffalo and cattle recorded sharing the same site on the same day were additionally highlighted. Analysis and mapping were done in R (R Core Team 2013) and Quantum GIS (QGIS Development Team 2015) with downloaded base maps (http://www.gadm.org).

### Interaction surveys

**FIGURE 1:**
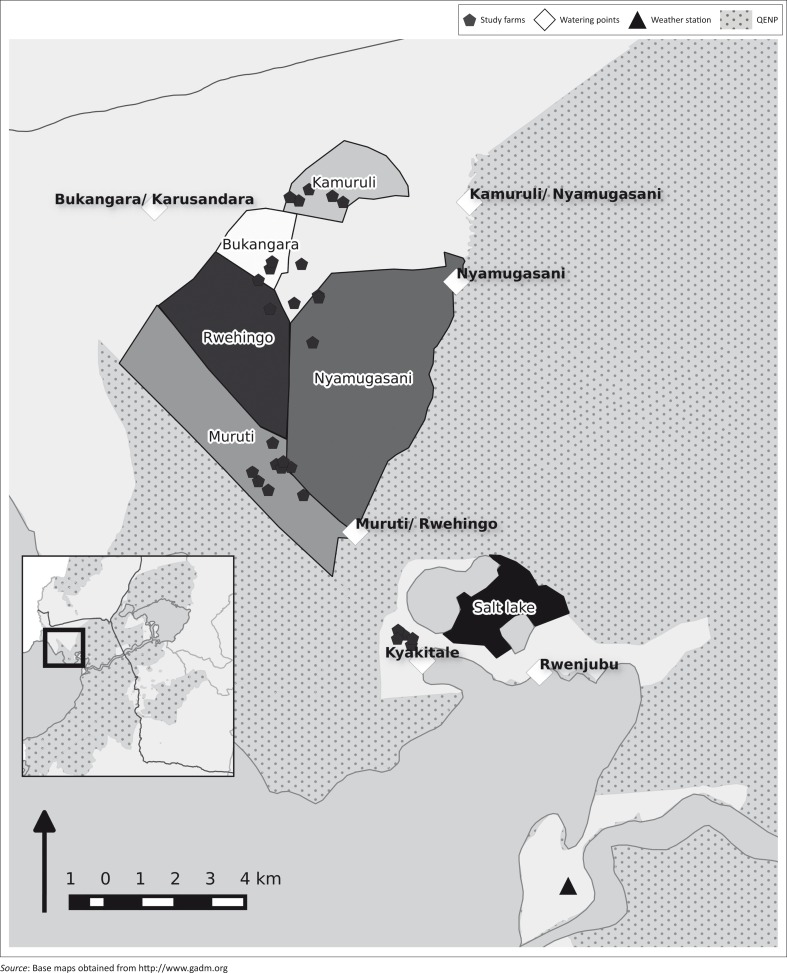
Study site in western Uganda showing location of study farms, communal grazing areas and watering sites. Inset: Queen Elizabeth National Park.

Farmers or employed herdsmen accompany their cattle herds to grazing and watering sites and return to enclose their animals near the homestead overnight. Twenty-five livestock owners from Nyakatonzi (northern border of QENP) and five livestock owners from Katwe Kabatoro (community within QENP) were identified to take part in a longitudinal survey recording daily wildlife sightings (Figure 1). These farmers, all members of the Muhumuza Nyakatonzi Cattle Keepers Association, were selected from within each parish in the study area. One member of the household was required to be able to read English. Verbal consent was obtained from the livestock owner after a prepared statement was read in the local language; participants could withdraw from the study at any time and were compensated for their participation. The livestock owners were asked to give monthly demographic information such as herd size, sales and losses of cattle. Daily information was requested on wildlife sightings by the livestock owners, noting the location of the sighting, the species involved and distance from domestic cattle, as well as grazing with other cattle herds, that is, how many cattle herds interact with his herd. This information was collected over 3 months from December 2014 to March 2015. An animal health technician conducted weekly visits to ensure questionnaires were completed. The survey period was preceded by a pilot survey for 1 month in November 2014 to test the questionnaires and the logistics and adjusted after consultation with the animal health technician involved.

Weather information was obtained from UWA for the weather station at Mweya outpost for 2002–2013 (Figure 1). Local regression smoothing was applied to the time-series data of the wildlife sightings to observe trends. The number and incidence of sightings (average sightings per farmer) were calculated and analysed in R statistical software. Mixed models were used to examine the relationship between the outcomes of sightings and distance to wildlife with the following variables: location type, herd size, location, distance to park, month and species where appropriate. Farmer ID was added as a random effect for all models. Herd size was divided into three categories (5–24, 25–75 and > 75). The distance between wildlife and livestock was condensed from five into two categories: ‘near’ (from: almost touched, 10 m) and ‘far’ (from: 50 m, 100 m and > 100 m). This was done to compensate for observer bias of distance, and the true ‘near’ distance is more likely to range from 5 m to 50 m. Bukangara was the reference location in the statistical models. The analysis focused on ungulates, which were comprised of African buffalo, Uganda kob, waterbuck and warthog, with additional individual focus on buffalo.

## Results

### Wildlife crime database

The wildlife crime database contained 5989 buffalo sightings and 426 cattle sightings for the years January 2006 – March 2014. The cattle were distributed along the northern borders of the park, whereas buffalo concentrations were higher to the south-west of Lake George and in the southern region of QENP, Ishasha sector. The spatial distribution of animals was similar by year and season; however, the total number of cattle sightings per year was variable.

**FIGURE 2:**
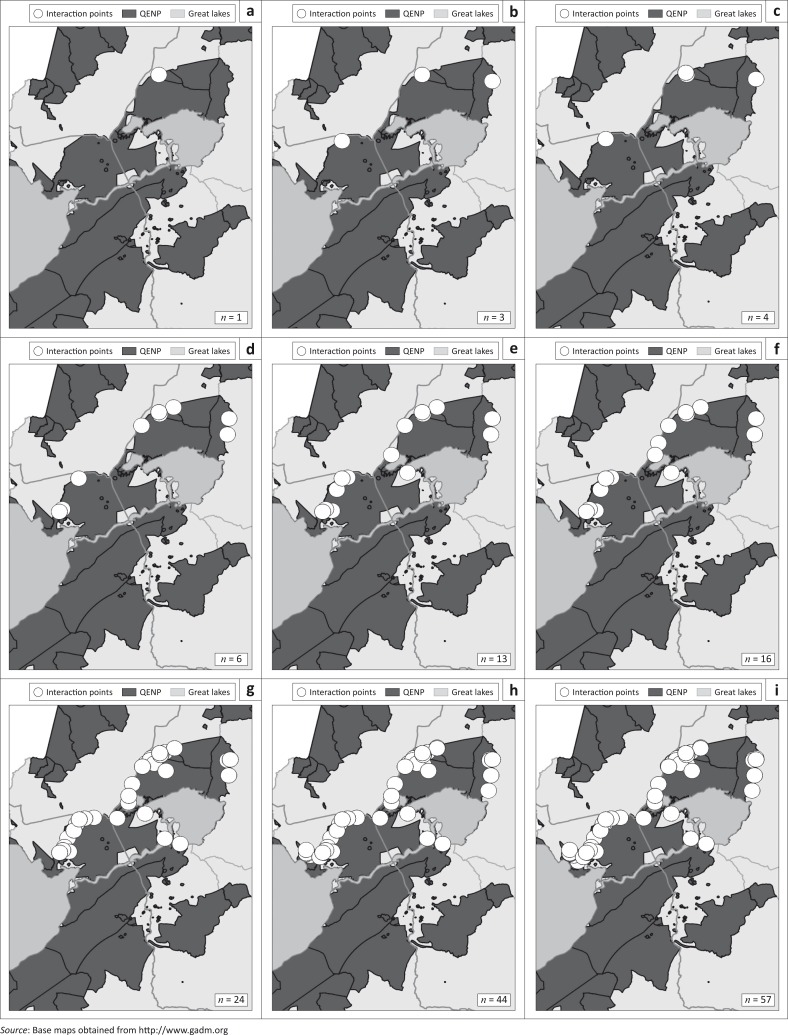
Queen Elizabeth National Park showing points of spatio-temporal overlap (*n*) between buffalo and cattle within (a) 2 weeks and 150 m, (b) 4 weeks and 150 m, (c) 6 weeks and 150 m, (d) 2 weeks and 300 m, (e) 4 weeks and 300 m, (f) 6 weeks and 300 m, (g) 2 weeks and 500 m, (h) 4 weeks and 500 m and (i) 6 weeks and 500 m.

The number of cattle and buffalo sightings within QENP per year is summarised in Table 1, highlighting the number of interactions within 4 weeks and 300 m. The locations and numbers of the wildlife-cattle interactions are shown in Figure 2. The areas where interactions occur follow the high density spatial distribution of the cattle along the northern border of QENP. There were 13 interactions at the 300 m–4 week limit (3.1% of cattle sightings) with 57 interactions at our maximum limits. Nine interactions were seen within 1 day at any distance < 500 m. The closest distance between species within 6 weeks was 101 m.

**TABLE 1:** Number of sightings per year of cattle and buffalo in the Queen Elizabeth National Park in the wildlife crimes database from Uganda Wildlife Authority.

Year	Cattle (*n*)	Buffalo (*n*)	Interactions (*n*) (4 weeks, 300 m)	Interactions per cattle sighting (%)
2006	100	608	0	0.0
2007	13	522	0	0.0
2008	34	606	1	2.9
2009	116	809	3	2.6
2010	84	925	5	6.0
2011	39	778	3	7.7
2012	29	828	0	0.0
2013	9	739	0	0.0
2014†	2	174	1	50.0
**Total**	**426**	**5989**	**13**	**3.1**

† January–March.

### Farmer interaction surveys

Thirty farmers participated in the longitudinal survey reporting wildlife-cattle interactions over a 3-month period from December 2014 until March 2015. The total cattle study population was reported to be 1869 adults and 381 calves on average per month. The average male:female ratio was 1:3, and the calf:adult ratio was 1:5. The median herd size was 38 animals (mean 62, range 5–237).

Farmers sent herds out to graze for an average of 10.9 hours (min–max 4.9–18.1 hours; s.d. 1.6) per day. The number of cattle herds seen mixing with other herds at the grazing points was on average five herds per day (median 4, s.d. 4.7). At the watering points, five herds on average were also seen per day (median 4, s.d. 4.1). Over the 3-month period, an average of six animals left a herd per month because of death, sale, theft or being lost during grazing. On average five animals entered a herd per month by buying in, births, gifts or being found whilst grazing. This resulted in an average loss of one animal per month per herd. More details of these gains and losses are shown in Tables 2 and 3.

**TABLE 2:** Average losses of cattle reported per herd per month.

Month	Total losses		Deaths		Sold		Lost		Theft	
	*n*	*n*/herd	*n*	*n*/herd	*n*	*n*/herd	*n*	*n*/herd	*n*	*n*/herd
Dec./Jan.	178	5.9	8	0.3	37	1.2	114	3.8	19	0.6
Jan./Feb.	162	5.4	12	0.4	51	1.7	74	2.5	25	0.8
Feb./Mar.	217	7.5	41	1.4	34	1.2	59	2.0	83	2.9

**TABLE 3:** Average gains of cattle reported per herd per month.

Month	Total gains		Births		Bought		Found		Gifts	
	*n*	*n*/herd	*n*	*n*/herd	*n*	*n*/herd	*n*	*n*/herd	*n*	*n*/herd
Dec./Jan.	181	6.0	29	1.0	2	0.1	145	4.8	5	0.2
Jan./Feb.	167	5.6	27	0.9	5	0.2	134	4.5	1	< 0.1
Feb./Mar.	112	3.9	21	0.7	7	0.2	83	2.9	1	< 0.1

**FIGURE 3:**
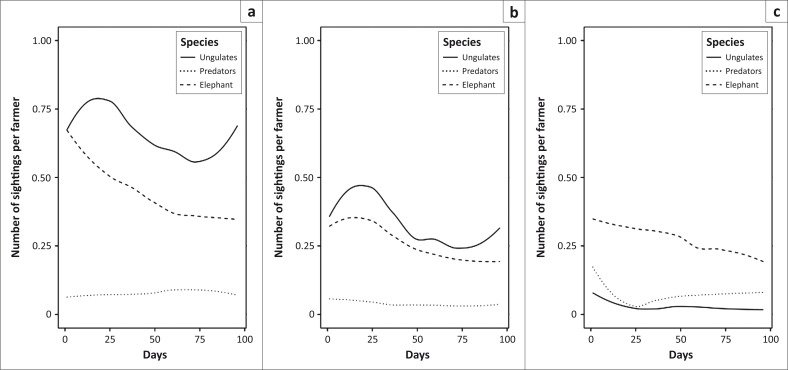
Number of daily sightings per farmer of elephant, predators and ungulates for the period December 2014–March 2015. (a) Grazing area, (b) watering point and (c) homestead.

**FIGURE 4:**
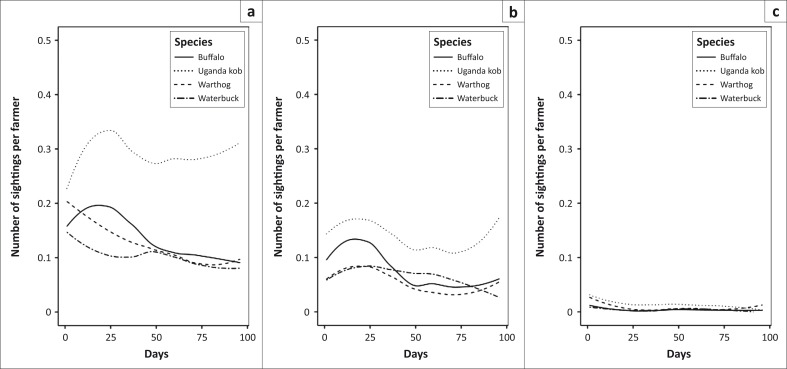
Number of sightings per farmer per ungulate type, plotted per day for the period December 2014–March 2015. (a) Grazing area, (b) watering point and (c) homestead.

**FIGURE 5:**
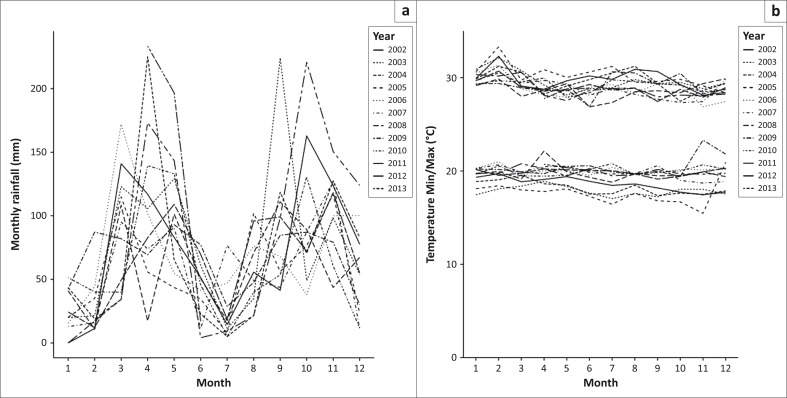
(a) Monthly rainfall and (b) mean monthly min–max temperature at Uganda Wildlife Authority Mweya weather station in Queen Elizabeth National Park for 2002–2013.

In total, there were 2744 daily reports from farmers covering the 3-month period from 24 December 2014 until 25 March 2015. Farmers reported wildlife sightings on 73.1% of farmer days. Ungulates, including buffalo, were seen by farmers on 52.7% of reported days (2901 total sightings), with a mean incidence of 1.04 (95% CI 0.97–1.11) animal sightings per farmer per day. Buffalo were seen on 18.8% of reported days at a rate of 0.22 (95% CI 0.20–0.25) sightings per farmer per day. In addition to ungulates, elephants were seen on 57.6% of days, predators (lion, leopard, hyena) on 13.2% of days (Figure 3) and other wildlife were mentioned in 9.2% of the reports. These included reports of hippopotamus, Nile crocodile, rabbit (most likely scrub hare) and unspecified snake and bird species. Ungulates were seen more frequently at grazing areas compared with watering points and homesteads. The total number of sightings decreased over the 3 months studied (Figure 4). Uganda kob (*n* = 1257) were the most commonly seen ungulate, followed by buffalo (*n* = 635), warthog (*n* = 525) and waterbuck (*n* = 484). The beginning of the rainy season usually falls within this time period (months 1–3 in Figure 5) based on data from the Mweya weather station.

Nyamugasani grazing and watering points were the areas most frequently used by farmers. Nyamugasani had the highest absolute number of ungulate sightings (52.1%) and buffalo sightings (51.7%). Relatively, Rwehingo grazing area (OR 2.1; 95% CI 1.5–3.1; *p* < 0.001), Nyamugasani grazing area (OR 1.4; 95% CI 1.0–1.9; *p* = 0.05) and grazing on multiple sites (OR 4.1; 95% CI 1.2–13.8; *p* = 0.02) were associated with greater odds of ungulate sightings compared with Bukangara, in the mixed univariate model (Table 4). When examining buffalo sightings, Rwehingo (OR = 1.8, 95% CI 1.1–2.9; *p* = 0.02) and multiple sites (OR 5.2, 95% CI 1.9–14.4; *p* = 0.002) had increased odds; however, there was no association with specific watering locations. Ungulates (*n* = 72, 2.6%) and buffalo (*n* = 10, 0.4%) were seldom reported close to the homesteads.

**TABLE 4:** Grazing and watering locations showing the area visited and the percentage of ungulate and buffalo sightings at each location.

Location	Grazing area				Watering point			
	Visited (*n*)	Sighting %		Visited (*n*)	Sighting %	
		Ungulate	Buffalo		Ungulate	Buffalo
Bukangara	253	41.5	10.7	296	27.7	7.1
Muruti	255	44.3	11.0	61	21.3	8.2
Nyamugasani	1183	47.0*	11.5	1645	28.2	9.3
Rwehingo	539	62.0***	19.3*	78	35.99	5.1
Salt Lake	36	36.1	11.1	0	0.0	0.0
Town Council	427	50.6	18.3	0	0.0	0.0
Kanyampara	0	0.0	0.0	121	43.0	16.5
Kyakitale	0	0.0	0.0	460	13.7	3.5
Other	31	6.5**	3.2	83	6.0**	2.4
Multiple sites	20	80.0*	45.0**	0	0.0	0.0
**Total**	**2744**	**49.4**	**14.1**	**2744**	**25.8**	**8.1**

*p*-value:

* < 0.05;

** < 0.01;

*** < 0.001; univariate model with farmer as a random effect.

There were greater odds of an ungulate sighting, within the first 2 months of the study or from farmers with larger herd sizes (OR 2.7), using the univariate analysis (Table 5). The evidence of a herd size effect was lost in multivariate models as there was irregular distribution of herd sizes amongst parishes. Muruti parish tended to have larger herds, and Kyakitale had smaller herds than average, although homesteads from these locations were closest to the park borders. Homesteads closer to the park were more likely to see ungulates with a 0.97 decrease in odds (95% CI 0.95–0.99) of a sighting per 100 m increase in straight-line distance to the QENP border (*p* < 0.01). The average distance of homesteads, where sightings took place, was 1100 m from the border of QENP, and where no sightings took place, the average distance was 2400 m.

**TABLE 5:** Risk factors associated with sightings of ungulates and buffalo reporting odds ratios for univariate mixed models with farmer as a random effect.

Risk factor	Reports (*n*)	Ungulate		Buffalo	
		Sightings (%)	OR	95% CI	Sightings (%)	OR	95% CI
**Herd size**							
5–24	898	53.0	1.0	-	13.9	1.0	-
25–75	1199	46.5	0.7	(0.3–1.5)	15.4	1.4	(0.6–3.2)
> 75	647	74.5	2.7	(1.1–6.5)*	31.7	3.7	(1.4–9.7)**
Month							
March	768	50.9	1.0	-	13.5	1.0	-
February	838	50.8	1.0	(0.8–1.2)	15.2	1.2	(0.9–1.6)
January	929	59.2	1.5	(1.2–1.8)***	24.4	2.4	(1.8–3.1)***
December	209	70.8	2.7	(1.9–3.9)***	27.3	2.8	(1.9–4.2)***

*p*-value:

* < 0.05;

** < 0.01;

*** < 0.001.

Analysing the subset of sightings, during the 3-month period, 14.7% of the ungulate sightings and 12.0% of the buffalo sightings were reported as ‘near’ distance to the cattle herd. Of these, buffalo were never reported as ‘almost touching’, and only *n* = 6/1612 (0.4%) ungulates (two Uganda kob, four waterbuck) were reportedly close enough to touch the livestock. An additional 31.1% of buffalo sightings were considered to be roughly 50 m away compared with 40.8% of the ungulate sightings. With the univariate mixed model, ungulates had greater odds of a ‘near’ distance sighting at the homestead (OR 2.0) (Table 6). Those with larger herd sizes were more likely to report a ‘near’ sighting with buffalo and ungulates. There was an inverse relationship between homestead distance from the park boundary and a ‘near’ sighting (OR 0.95, 0.93–0.97 per 100 m).

**TABLE 6:** Risk factors associated with distance (near vs. far) of cattle from wildlife reported for ungulates and buffalo including odds ratios for univariate mixed models with farmer as a random effect.

Risk factor	Ungulate				Buffalo			
	Reports (*n*)	Near distance (%)	OR	95% CI	Reports (*n*)	Near distance (%)	OR	95% CI
**Location type**								
Grazing area	1879	13.9	1.0	-	399	9.8	1.0	-
Watering area	945	14.8	1.1	(0.9–1.4)	226	15.5	1.4	(0.8–2.5)
Homestead	77	32.5	2.0	(1.1–3.6)**	10	20.0	1.8	(0.3–12.1)
**Herd size**								
5–24	834	10.3	1.0	-	176	1.7	1.0	-
25–75	1040	10.5	1.3	(0.4–3.9)	227	2.6	1.5	(0.3–7.3)
> 75	1027	22.6	3.7	(1.1–12.8)*	232	28.9	21.8	(5.1–92.5)***
**Ungulates**								
Buffalo	635	12.0	1.0	-	-	-	-	-
Uganda kob	1257	14.9	1.6	(1.2–2.1)**	-	-	-	-
Warthog	525	16.4	1.4	(1.2–2.0)***	-	-	-	-
Waterbuck	484	16.1	1.5	(1.0–2.1)*	-	-	-	-

*p*-value:

* < 0.05;

** < 0.01;

*** < 0.001.

## Discussion

This study described reports of wildlife proximity to livestock around QENP using an existing wildlife sightings database as well as daily sighting reports from livestock owners. The value of wildlife sighting reports is limited in that absence of a reported sighting is not necessarily absence of an animal. A benefit of the longitudinal livestock owners study is that herdsmen remain with the cattle for most of the day, which would increase the likelihood of a reported sighting. We reported a larger number of sightings compared with similar research on the border of the Kruger National Park in South Africa (Brahmbhatt et al. 2012). However, only fenced areas of the park were included in that study implying that contacts were reported when wildlife or livestock crossed this barrier, whereas free movement is possible at QENP.

The daily reporting of sightings by livestock owners minimised recall bias, although there was a risk of reporting fatigue as the study progressed. Employment of a field technician performing weekly visits encouraged completion of the questionnaires by livestock owners. The tapering of wildlife sightings over time may still be a consequence of fatigue, but the levels of Uganda kob sightings did not differ significantly by month on Pearson’s chi-squared test (*p* = 0.1). This supports the outcome that the decrease of total sightings is a real finding. This may be related to the onset of the rainy season and would need to be confirmed with an extended study across seasons. Ryan, Knechtel and Getz (2006) showed a seasonal change in the home range of buffalo related to water availability and quality of pastures, as well as changes in herd size, which may account for the decreased number of sightings over time. Although water availability may not be a factor, a perennial river is the main water source on the QENP boundary. Additionally, in late January, large areas of QENP were subject to wildfires, which probably influenced the grazing patterns of the wildlife, including areas adjacent to our study site. Animals may have moved deeper into the park in search of grazing until there was sufficient regrowth in the burnt areas.

Although elephant and predator sightings were not the focus of this study from a disease point of view, their sightings, especially the high number close to the homesteads, were associated with destruction of property and loss of livestock. These species are a source of conflict between the wildlife authority and community farmers, who do not feel appropriately compensated for their losses (B. Sunday, pers. comm., November 2014).

The role of ungulates, besides buffalo, in the transmission and maintenance of BTB is not known in the wildlife of QENP. BTB was confirmed in Uganda kob for the first time in QENP in 2011, after a cluster of mortalities was investigated (PREDICT Consortium 2014; B. Ssebide, pers. comm., March 2015). In warthog, *M. bovis* was isolated from a necropsy in 1997 (Kalema-Zikusoka et al. 2005) confirming the ongoing infection in this species since previously reported by Woodford (1982b). Considering the close contact these species have with buffalo and cattle, this association should be further explored. Warthog may contribute to disease in a similar manner as wild boar in Europe (Hardstaff et al. 2013; Palmer 2013) or Uganda kob likewise to the Kafue lechwe antelope in Zambia (Munyeme & Munang’andu 2011). Nevertheless, environmental conditions differ and the wetlands favoured by Kafue lechwe may aid survival and spread of *M. bovis* in its habitat.

Close contact does not necessarily equate to an effective transmission event, and the potential for disease transmission across species is likely influenced by a number of factors. Firstly, the variability in infectiousness of the animals because of disease progression and species will impact transmission. In an experimental study in South Africa, it was suggested that African buffalo do not commonly shed high quantities of *M. bovis* in nasal and oral secretions and are unlikely to transmit infection through water in free range conditions (Michel et al. 2007). This, however, did not preclude the possibility of the spread of other mycobacterial species. Conversely, in cattle, nasal excretion of *M. bovis* was seen more commonly (Menzies & Neill 2000). The tendency to graze cattle illegally within QENP would therefore allow for contamination of the wildlife grazing areas as well as the communal pastures and watering points.

A second factor influencing effective transmission is survival of the pathogens dependant on environmental conditions. We used up to 6 weeks as an indicator of survival time of *M. bovis* as shown by Tanner and Michel (1999), but this may be an overestimate. Jackson, De Lisle and Morris (1995) showed an inverse relationship of bacterial survival with high temperature implying that under the hot conditions found at our study site (20 °C – 30 °C), *M. bovis* is unlikely to survive beyond 4 weeks unless in the shade (Duffield & Young 1984). It should also be considered that for oral transmission to take place, a susceptible animal must graze the specific location where an infectious animal had been within this time frame. Under extensive grazing conditions, this is likely to be an uncommon occurrence.

The possibility of infection transmission will also be influenced by the movement and distribution of animals regulating direct and indirect contact rates. There were limitations to the distances reported in this study, as they were an approximation. Animals may have moved around the recorded point and contacts between species may have gone unnoticed. No buffalo were reported to be in direct contact with cattle in the livestock owner reports and no buffalo came within 100 m of cattle in the ranger reports. In a study by Miguel et al. (2013) using GPS collared animals, direct contact between buffalo and livestock was also limited. For aerosol transmission, few bacilli carried in droplets can transmit BTB infection (Neill, Bryson & Pollock 2001). Close contact between cattle herds occurs with congestion at the entrance to watering points and kraaling at night which would encourage droplet transmission between cattle (Gannon, Hayes & Roe 2007), whereas this close contact was rare between wildlife and cattle. Additionally, Pollock et al. (2006) reviewed evidence indicating that in outdoor conditions, transmission rates for BTB were low.

The prevalence of BTB in individual cattle around QENP is estimated at 2.8% (95% CI 1.5–5.4) (Meunier, unpublished data), whereas in buffalo BTB is reported at 21.6% (Kalema-Zikusoka et al. 2005). Considering the lack of control measures in cattle for BTB, it is not unreasonable to expect higher levels of BTB infection in local cattle if a high frequency of transmission from buffalo was taking place. Alternately, Ankole cattle, the widely kept local breed, may be less susceptible than expected to BTB (Ameni et al. 2007). In South Africa, there is evidence of the same spoligotypes of BTB present in both wildlife and livestock (Hlokwe et al. 2014). Identification of the strain types involved in infection of wildlife and livestock bordering QENP could give a better indication if inter-species spread is occurring.

## Conclusion

This study quantified reports of wildlife seen near to livestock around QENP showing that inter-species transmission of BTB infection is possible and showed feasible high-risk areas for interactions on the border of QENP. Sharing of grazing and water resources occurred frequently, which would favour environmental transmission. In contrast, infrequent close contact between species occurred which could possibly facilitate aerosol transmission. Although considering the environmental conditions, host determinants and pathogen factors, transmission of BTB between species is likely a rare spillover event. Other diseases, particularly those with tick vectors, could be easily transmitted within the time frames and distances reported in our study (Caron et al. 2013). Regarding the high number of sales in cattle herds, disease introductions in cattle from other livestock are likely to be a high-risk event, even though farmers may first implicate wildlife as a source of infection. Disease control at the point of movement and sale of cattle will presumably have a greater impact on the spread of BTB in livestock than controlling the interface between wildlife and cattle in a situation where eradication is not being considered. However, this intervention does not address the spread of the disease within wildlife. It is unknown what role other wildlife such as the Uganda kob, waterbuck and warthog play in infection transmission within this system, although they were regularly sighted near cattle and homesteads.
